# A catalyst for change

**DOI:** 10.7554/eLife.71583

**Published:** 2021-07-02

**Authors:** Chrissy Stachl

**Affiliations:** 1 NSF Center for Genetically Encoded Materials, University of California, BerkeleyBerkeley, CAUnited States

**Keywords:** research culture, sparks of change, equity, diversity, inclusion

## Abstract

Disillusioned with conducting chemistry experiments in a basement, a new graduate student decides instead to dedicate her PhD to improving the culture of her research environment.

Graduate students, postdoctoral researchers, and faculty had started to arrive in large numbers, quickly filling up the auditorium for our departmental town hall. Facing the growing crowd, I could feel the butterflies in my stomach and my heart starting to race. A fellow PhD candidate and I were here to present the results of a department-wide survey assessing how the community felt about their research environment. We were going to ask our audience to decide on how to best tackle the issues we had uncovered. Before this day, only faculty had ever led our town halls — a dynamic which left many in the audience feeling dissatisfied. Now the incoming department chair had just handed us the microphone.

**Figure 1:**
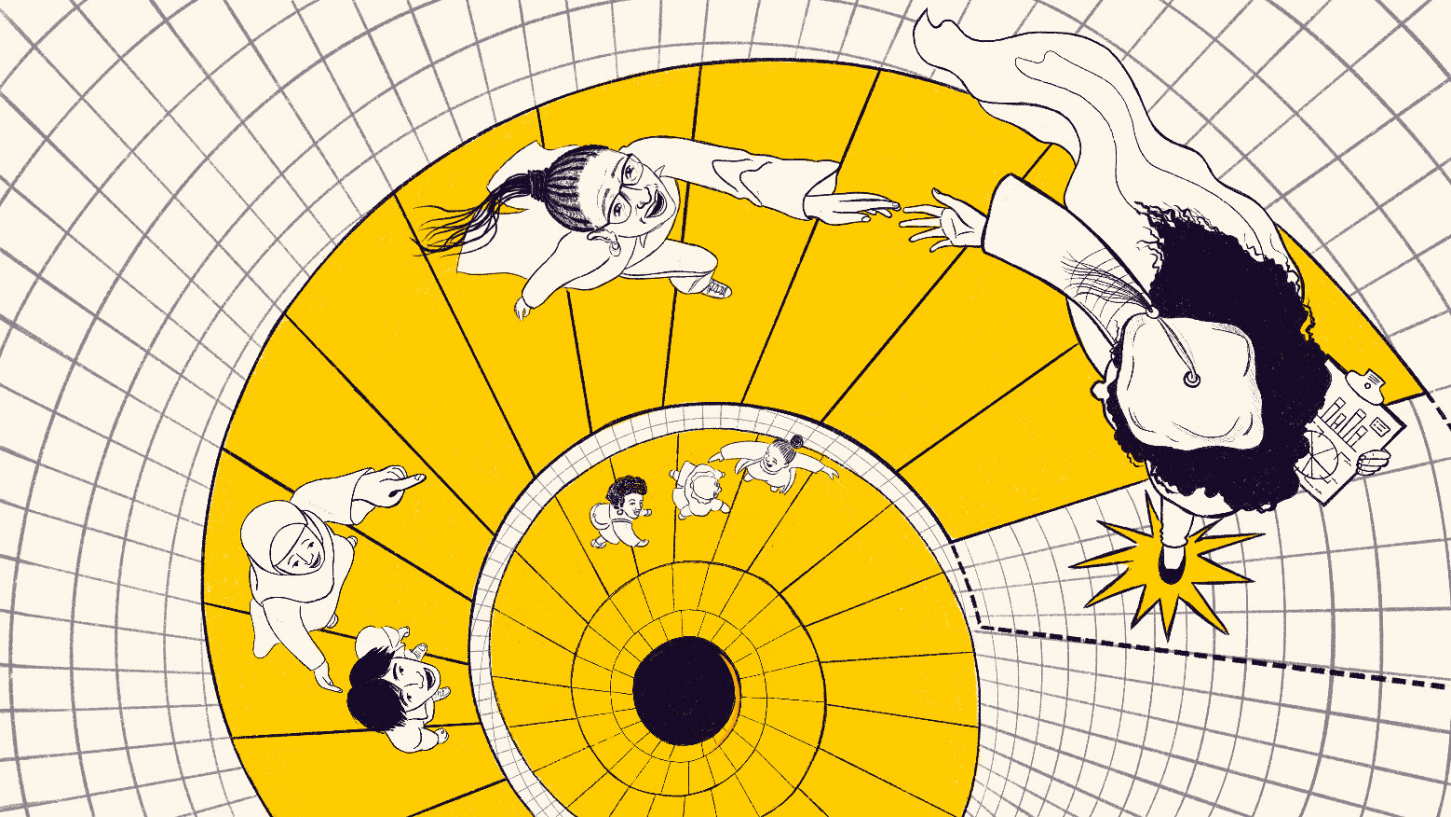
Data in hand, a PhD student challenged the status quo in her department and helped to improve its culture for those who came next.

IMAGE CREDIT: Carys Boughton; http://www.carysboughton.com (CC BY 4.0).

Standing on that stage is not what I expected when I started my PhD in the Department of Chemistry at the University of California, Berkeley, three years before. Yet leading up to this point, my experiences within academia had made my love for science feel more like a battle than a journey. I was raised by high-achieving, confident Latin women, and grew up immersed in a tight-knit community that thrived on diversity, respect, and unwavering support. So it was not until I left home to start my undergraduate degree that I was made aware that my ethnicity was ‘underrepresented’, and that it made me an outsider — particularly within academia. Having to adapt to a culture that made me feel powerless and isolated was heartbreaking, but it also fueled my determination to understand and combat the underrepresentation of women and other historically marginalised groups in science. My feelings of isolation worsened as I entered graduate school, and I found myself unsupported by my research advisor.

As one of only a few Latin women in a department of over 400 graduate students, I frequently sought out spaces where I might feel more accepted and welcome. Eventually, I became involved in our chemistry graduate life committee and began to realize that other students also felt they were not receiving the support we all needed. At the same time, it became clear that previous efforts to improve the department climate had not gained much traction, and that only quantitative data would convince the administration to consider the mentorship, well-being, and inclusion issues faced by our graduate students.

...only quantitative data would convince the administration to consider the mentorship, well-being, and inclusion issues faced by our graduate students.

So there we were, standing in front of the crowd, data in hand. Getting to this point had taken us two years, and we had collaborated with everyone willing to help — from faculty and staff to students and the administration. It had paid off: over 40% of the department had responded to the survey. The results revealed, for instance, that graduate students and postdocs wanted (but were reluctant) to ask their advisors for emotional support and non-research advice, while faculty did not know where to find resources to provide such help. Overall, the community voiced their desire for more spaces in which they could share concerns and perspectives, and be heard without judgement. Respondents also strongly agreed that diversity needed to improve across all academic levels, and wished to be educated about biases and behaviors that negatively affect women and people of color.

Once we had shared our findings, we split the attendees into small student-led groups where they were invited to discuss one of three topics and their corresponding, pre-determined questions: diversity and inclusion, mental health and work environment, or mentorship and faculty-student interactions. The goal for each group was to use our data to find concrete ways to improve the culture of our department. Soon, the hall resonated with the intrigued and excited chatter of groups working together, taking advantage of this new space for collaboration and discussion. The veil of anxiety lifted off me; elation took its place.

In the following months I threw myself into spearheading many of the interventions our community had come up with, and helped to ensure that they became a reality. A monthly diversity and inclusion focus group was created —﻿ a safe space bringing together faculty, students, staff, and postdocs to discuss issues within the department. Trainee feedback started to be considered for faculty hiring, with graduate students being given the opportunity to assess candidates in a systematic way. For example, each applicant is asked to discuss their mentoring practices, how they deal with conflict, their plan to contribute to diversity and inclusion in the department, and how they plan to support their trainee’s well-being. Discussions about mental health and cultural adaptation, training on how to respond to sexual violence and harassment, as well as information about leaving the program or switching research groups were incorporated into the graduate program and publicized to new students during orientation. The interventions were designed to eliminate stigmas that have typically shrouded conversations about equity, inclusion, and well-being, and to actively acknowledge and prioritize the needs of trainees from historically marginalized groups in science. This improved communication also spread to research groups, paving the way for more holistic and tailored mentoring between faculty and trainees.

As I became increasingly enamored with the tangible, positive impacts I was making within my department, the motivation for my physical chemistry research eroded. Around me, the cracks that Donald Trump’s presidency was creating in society and in my own communities were exposing, again, the systemic oppression that plagues my country. I could not stop asking myself: why am I spending every day in a windowless basement, working on an unfulfilling scientific project that I did not choose, when the world so desperately needs passionate individuals fighting for equity and justice? With the support of senior leadership, I made the decision to leave physical chemistry behind, switch research groups, and use the skills I gathered during my training to focus my dissertation on understanding and improving the climate and culture of my department.

The survey has been run again every spring since that first reformatted town hall meeting in 2018. Collectively, the data show that people are aware of the interventions we put in place, and that the perception of the department’s climate had steadily improved. For instance, graduate students and postdoctoral scholars increasingly felt that the department sufficiently discussed and acted towards improving diversity and inclusion. In parallel, I trained a new team of students to keep moving forward with this work and ensuring it remains a priority.

With this job done, I have now completed my PhD. Looking back, I feel awestruck that I had the opportunity to challenge the status quo in my research community. In doing so, I re-discovered my own sense of belonging, found a new way to thrive in academia, and helped to make my community more inclusive for those coming after me.

## Share your experiences

This article is a Sparks of Change column, where people around the world share moments that illustrate how research culture is or should be changing. Have an interesting story to tell? See what we’re looking for and the best ways to get in touch here.

